# BECTS Substate Classification by Granger Causality Density Based Support Vector Machine Model

**DOI:** 10.3389/fneur.2019.01201

**Published:** 2019-11-14

**Authors:** Xi-Jian Dai, Qiang Xu, Jianping Hu, QiRui Zhang, Yin Xu, Zhiqiang Zhang, Guangming Lu

**Affiliations:** ^1^Department of Medical Imaging, Jinling Hospital, Medical School of Nanjing University, Nanjing, China; ^2^Shenzhen Mental Health Centre, Shenzhen Kangning Hospital, Shenzhen, China; ^3^Sleep Assessment Unit, Department of Psychiatry, Faculty of Medicine, Chinese University of Hong Kong, Hong Kong, China

**Keywords:** benign epilepsy with centrotemporal spikes, granger causality density, seizure disorder, support vector machine, classification, prediction

## Abstract

**Objectives:** To investigate the performance of substate classification of children with benign epilepsy with centrotemporal spikes (BECTS) by granger causality density (GCD) based support vector machine (SVM) model.

**Methods:** Forty-two children with BECTS (21 females, 21 males; mean age, 8.6 ± 1.96 years) were classified into interictal epileptic discharges (IEDs; 11 females, 10 males) and non-IEDs (10 females, 11 males) substates depending on presence of central-temporal spikes or not. GCD was calculated on four metrics, including inflow, outflow, total-flow (inflow + outflow) and int-flow (inflow – outflow) connectivity. SVM classifier was applied to discriminate the two substates.

**Results:** The Rolandic area, caudate, dorsal attention network, visual cortex, language networks, and cerebellum had discriminative effect on distinguishing the two substates. Relative to each of the four GCD metrics, using combined metrics could reach up the classification performance (best value; AUC, 0.928; accuracy rate, 90.83%; sensitivity, 90%; specificity, 95%), especially for the combinations with more than three GCD metrics. Specially, combined the inflow, outflow and int-flow metric received the best classification performance with the highest AUC value, classification accuracy and specificity. Furthermore, the GCD-SVM model received good and stable classification performance across 14 dimension reduced data sets.

**Conclusions:** The GCD-SVM model could be used for BECTS substate classification, which might have the potential to provide a promising model for IEDs detection. This may help assist clinicians for administer drugs and prognosis evaluation.

## Introduction

Recently, functional neuroimaging methods have been widely used to describe functional network changes and the relationships among different brain networks in diseases. The most widely used method is functional connectivity, which involves calculation of the correlation of time courses between one brain region and each of the rest of brain regions. Another method, functional connectivity density, which unbiasedly measures the functional connectivity strength over the whole brain, could reflect the communication amounts among brain regions (1). However, both methods cannot reflect the directed information flow among different brain regions. Although granger causality analysis fulfills this requirement (2–4), it is based on priori hypotheses of definition of regions of interest. Reliable and accepted methods are needed. A new method, called granger causality density (GCD), aggregates conditional information sets according to community organization using weighted connectivity density map, reflecting the average effect connectivity strength between each voxel and the rest of voxels of whole brain. The proposed GCD analysis could avoid redundancy and overfitting, which makes it suitable for neuroimaging data analysis, and even for high-dimensional and short dataset. Furthermore, this method could provide an opportunity for unbiased searches abnormalities within the entire connectivity matrix without any priori hypotheses, and reflect the directed information flow among different brain networks from voxel levels. However, it has not been applied into any diseases.

Group-based methods are not helpful in inferring specific clinical outcomes for individual patients. Current functional MRI researchers mainly focus on describing group differences between subject classes (knowing the label of each subject), which cannot be classified across different types of subjects. Therefore, for the purpose of individual classification, a desirable method would be one that can compare a single subject's scan to a group. The interictal epileptiform discharges (IEDs) detection remains a challenging problem for simultaneous EEG-fMRI examination because of the absence of long-term data recordings, which are not like the single EEG data recordings, and the insufficient data recordings cannot be used for training and testing (5, 6). Support vector machine (SVM) classifier recognition algorithm is a sensitive neuroimaging bioindicator and efficient feature-selection method. The SVM can train a classifier to classify the label of an unseen subject by taking multiple features into account jointly. There is a growing findings in data-analytic modeling for detection and seizure prediction from intracranial EEG recording (5–8). Seizure prediction has the potential to transform the management of patients with epilepsy by administering preemptive clinical therapies (such as neuromodulation, drugs) and patient warnings (9). The SVM-based model can help us to explore the voxel features or target brain regions with a high contribution to classification or prediction, and has been successfully applied to EEG data (6), but whether the functional MRI data could be used for IEDs prediction or classification left largely unknown. Epilepsy is a disease with brain network disorders (10, 11). Observation of the directed information flow propagation of the IEDs is one of the most important clinical purposes of epilepsy. Benign childhood epilepsy with central-temporal spikes (BECTS), also known as Rolandic epilepsy, is the most common type of idiopathic epilepsy in childhood. BECTS is a highly prevalent idiopathic epilepsy, affecting about 15.7% of epilepsy with 75% starting between 7 and 10 years (12, 13). Children's brains are developmentally immature and the nerve excitabilities are high, and therefore the children are more susceptible to epilepsy due to internal and external factors (13), which makes the BECTS considered to be an ideal model to describe the directed information flow differences in brain networks between IEDs and non-IEDs substates.

Seizure refers to the transformation of normal neurons into abnormally high excitatory and super synchronous electrical activities, resulting in recurrent episodes of transient seizures and brain dysfunction (14, 15), which is thought to be caused by an imbalance between excitation and inhibition (14). It has been suggested that brain excitation/inhibition imbalance is an important mechanism for leading to an overexcited epilepsy-related networks (16–18). The onset of the IEDs may break the excitatory/inhibition balance of neurons, leading to high excitatory and super synchronous electrical activities of epilepsy-related neural networks, and resulting in an imbalance of directed information flow among these networks. Therefore, we hypothesized that the IEDs substate may differ from the non-IEDs substate. Since the spread of epileptic activity is characterized by input and output information flow (19–24), combining the input and output information flow features may reach up the classification performances. To address these hypotheses, the present study is the first to apply the GCD-SVM model to explore a highly sensitive neuroimaging biomarker for BECTS substates classification. Previous studies have found that the GCA method may help patients with epilepsy for substate classification to discriminate between interictal and ictal status (25), which has important guiding significance for the decision-making of intraoperative surgical procedures. The present study may provide a potential biomarker to discriminate the BECTS having or not having the IEDs, and evaluate the possible mechanism of brain damage caused by the differences, which may be helpful to build an imaging model to predict remission and prognosis of BECTS, and have the potential to assist clinicians for clinical administration and monitoring the efficacy of disease-modifying therapies.

## Materials and Methods

### Subjects

Forty-two children with BECTS (21 females, 21 males; mean age, 8.6 ± 1.96 years) underwent simultaneous EEG-fMRI examination. The BECTS were classified into IEDs (11 females, 10 males; 5~12 years) and non-IEDs (10 females, 11 males; 6~12 years) substates depending on the presence of central-temporal spikes or not from the EEG-fMRI examination.

Inclusion criteria were as follows: (a) clinical and EEG findings indicative of BECTS, (b) aged between 5 and 17 years, (c) attending school regularly for education, (d) no developmental disabilities, (e) full-scale intelligence quotient of more than 70, and (f) no history of addictions or neurological diseases other than epilepsy. Patients received diagnoses on the basis of all available clinical and EEG data according to the following criteria: (a) International League Against Epilepsy classification (26) and current literature (13); (b) presence of simple partial, often facial, and motor or tonic-clonic seizures during sleep; and (c) spike waves in centrotemporal regions.

Exclusion criteria were (a) pathological focal brain lesions on T1-weighted and T2-weighted fluid-attenuated inversion-recovery MR images, (b) falling asleep during the MRI session (assessed by means of self-report and occurrence of sleep waves in simultaneously recorded EEG data), (c) head motion with more than 1.5 mm in translation or 1.5° in rotation, (d) age <5 years, (e) any history of significant head trauma or loss of consciousness >30 min, (f) any foreign implants, and (g) any history of neurological disorders or psychiatric illnesses.

This study was approved by Medical Research Ethical Committee of our Hospital in accordance with the Declaration of Helsinki and written informed consent was obtained from all subjects and their guardians.

### Simultaneous EEG and Functional MR Imaging Acquisition

During the fMRI data acquisition, the EEG data were continuously recorded with an MR-compatible recording system (Brain Products, Gilching, Germany). A total of 32 channels MR compatible Ag/AgCl electrodes (Brain Product, Munich, Germany) with reference at the electrode FCz and electrocardiography were attached to the scalp and connected to a Brain map amplifier. EEG data sets were processed offline to remove MR and ballistocardiographic artifacts (Brain Vision Analyzer 2.0; Brain Products, Munich, Germany). The EEG data were transmitted via an optic fiber cable from the amplifier placed inside the scanner room to a computer outside. The IEDs were marked on artifact-removed EEG by an experienced electroencephalographer and a neurologist.

Functional and structural imaging data were acquired with a clinical 3-Tesla MRI scanner (SIEMENS Trio Tim, Erlangen, Germany) with a standard eight-channel head coil. A total of 176 high-resolution T1-weighted anatomical slices were acquired with a three-dimensional magnetization prepared rapid-gradient-echo sequence in a sagittal orientation (repetition time = 2,300 ms, echo time = 2.98 ms, thickness = 1.0 mm, matrix = 256 × 256, field of view = 256 mm × 256 mm, flip angle = 9^0^). A total of 250 functional images were acquired using a single-shot Gradient-Recalled Echo-Planar Imaging pulse sequence (repetition time = 2,000 ms, echo time = 30 ms, thickness = 4.0 mm, inter-slice gap = 1.2 mm, field of view = 220 mm × 220 mm, matrix = 64 × 64, flip angle 90°, 30 transverse slices). The scan time of the functional data was 8 min and 10 s.

### GCD Data Processing

#### Data Preprocessing

The first 10 time points of the functional images were discarded due to the possible instability of initial MRI signal and inadaptation to the scanning environment. The remaining data were entered into pre-processing by Data Processing & Analysis for Brain Imaging (DPABI 2.1, http://rfmri.org/DPABI) toolbox, including the steps of data format transformation, slice timing, head motion correction, spatial normalization, and spatial smoothed using a Gaussian kernel of 8 × 8 × 8 mm^3^ full-width at half-maximum. Participants with more than 1.5 mm maximum translation in x, y, or z directions and/or 1.5° degree of motion rotation were removed.

To limit the impact of micro-movements artifacts, we implemented a “head motion scrubbing regressors” procedure as part of data preprocessing. An estimate of head motion at each time point was calculated as frame-wise displacement (FD) using Friston 24 head motion parameters procedure. The Friston 24 head motion parameter model was used to regress out the head motion effects. Images with FD >0.5 mm were removed and replaced by a linear interpolation. Linear regression was applied to remove other sources of possible spurious covariates, including the global mean signal, white matter, and cerebrospinal fluid signal. After the head-motion correction, the remaining images were spatially normalized to Montreal Neurological Institute (MNI) space and re-sampled at a resolution of 3 × 3 × 3 mm^3^. The time series for each voxel were further linearly detrended and temporally band-pass filtered (0.01–0.1 Hz).

#### Voxel-Based GCD Analysis

It is based on the idea that, given two time series of two voxels of *x* and *y*, if knowing the past of *y* is useful for predicting the future of *x*, then *y* must have a causal influence on *x*. The auto-regression model of the granger causality influence between the two time series of *x* and *y* variables were defined as follows:

$$\begin{array}{l} y_{t}={\text{a}}_{0}+{\text{a}}_{1}y_{\text{t-1}}+{\text{a}}_{2}y_{\text{t-2}}+...+{\text{a}}_{\text{m}}y_{\text{t-m}}+\;{\text{e}}_{1};\\ y_{t}={\text{a}}_{0}+{\text{a}}_{1}y_{\text{t-1}}+{\text{a}}_{2}y_{\text{t-2}}+...+{\text{a}}_{\text{m}}{\text{y}}_{\text{t-m}}+{\text{b}}_{\text{d}}{\text{x}}_{\text{t-d}}\\ \;+\;...+b_{\text{f}}x_{\text{t-f}}+\;{\text{e}}_{2}; \end{array}$$

The lagged value of d represents the earliest one in the significant time point of the *x(n)* variable, and *f* represents the closest one. Accordingly, the lagged value of m represents the earliest one of the *y(n)* variable.

*The* e_1_ represents the estimate residual of the autoregressive models of the time series of *x*(*n*), and the e_2_ represents the estimate residual of *y*(*n*) after adding the time series of *x*(*n*). Similarly, the definition of the variable of h_1_ and h_2_.

$$\begin{array}{l} \text{Generally},\text{ residual }{\text{e}}_{\left(\text{t}\right)}:\;|{\text{e}}_{1}|>=|{\text{e}}_{2}|; \end{array}$$

If *x* has a causal influence on *y*, the influence is defined as: F_x−>y_ = ln(|e_1_|/|e_2_|);

Similarly, the F_y−>x_ means y has a causal influence on *x*, and is defined as: F_y→*x*_ = ln(|h_1_|/|h_2_|).

The GCD algorithm has been improved based on the granger causality analysis algorithm. The GCD algorithm takes any one voxel of the brain voxels to define its time series as x, and the time series of the rest voxels are defined as y. Then, the linear direct influence of x on y (F_x−>y_) and the linear direct influence of y on x (F_y−>x_) were calculated voxel by voxel across the whole brain.

The F_x−>y_ value means output information flow from the targeted voxel (x) to whole brain voxels (y), and the F_y−>x_ means input information flow to the targeted voxel (x) from rest whole brain voxels (y). For the whole brain voxels, a series of F_x−>y_ and F_y−>x_ values are achieved, which reflects the output and input causal effective connectivity, respectively.

The density map of output causal influence of x variable on y variable is defined by the summation of the F_x−>y_ values (the threshold was defined as *p* < 0.05), namely outflow connectivity. Similar definition of the density map of input influence of y variable on x variable (F_y−>x_), namely inflow connectivity.

Considering that the graph GCD is directed, all topological properties are calculated on four metrics, including inflow, outflow, total-flow (inflow + outflow) and int-flow (inflow − outflow) connectivity.

The total-flow connectivity is defined as the combined effects of inflow and outflow connectivity. The int-flow connectivity is defined as the differences between the inflow connectivity and the outflow connectivity (F_y−>x_ − F_x−>y_), which identify nodes that have distinctive causal effects on network dynamics. Specifically, a node with a relatively high negative int-flow connectivity is regarded as more causal sources (driven effect), whereas a node with a relatively high positive int-flow connectivity is more causal targets (target effect).

### Voxel-Based Analysis for Each GCD Metric

**Figure 1:**
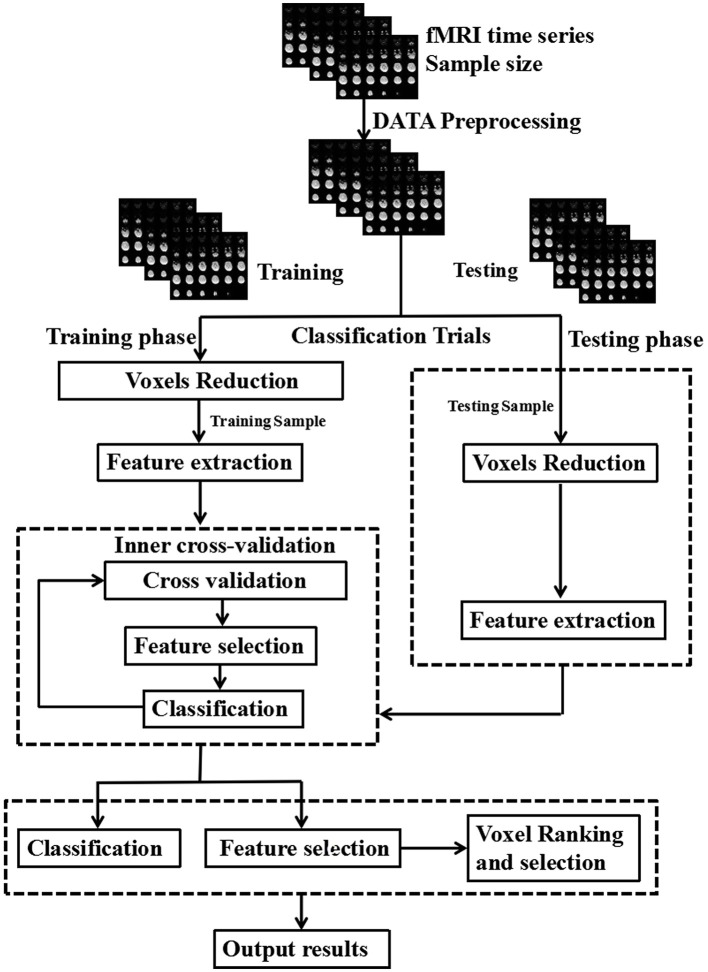
Schematic diagram overview of machine learning classification framework. The inner cross-validation was used to determine the optimal number of features and the outer cross-validation was employed to estimate the classification performance.

The LIBSVM toolbox (http://www.csie.ntu.edu.tw/~cjlin/libsvm/) was used to perform the classifications. Principal component analysis was used for dimension reduction. Finally, a linear SVM was used for images training and testing (Figure 1). Each image is treated as a point in a high dimensional space (space dimension = number of voxels in the image). In the present study, we classified the images into two classes (here, IEDs and non-IEDs substates) to find a potential separating hyperplane or decision boundary. The GCD images were entered into the classification procedure which consists of training phase and testing phase. A leave-one-out cross-validation test was used to evaluate the classification accuracy of the SVM classifier. The clusters with higher than 70% classification accuracy and contiguous voxels of more than 5 voxels were considered as accuracies. The resulting spatial map at each voxel with higher than 70% classification accuracy was used to find brain regions that exhibited differences between-groups.

### Classifier Performance of Combinations With Multiple GCD Metrics

The classification accuracy of the combinations with more than two GCD metrics were calculated using the linear SVM classifier. Multivariate pattern analysis (MVPA) technique was used to extract features, as the input of pattern analysis. The classifier is trained by providing examples of the form <x, c>, where x represents a spatial pattern (number of voxels in the image; here is pre-selected features of GCD images) and c represents the class label (here, IEDs and non-IEDs substates). To identify the set of voxels with highest discriminative power, SVM recursive feature elimination (SVM-RFE) was applied (27). The SVM-RFE classifier is repeatedly trained, and at each iteration, a feature-ranking criterion is used to remove a subset of the least informative features. Once the decision function is learned from the training data, it can be used to classify the class of a new test sample. The parameter (C) controls the trade-off between zero training errors and misclassifications, which was fixed at C = 1 for all cases (default value).

The performance of the classifier was validated by the commonly used 5-fold cross validation approach, which tested the robustness of the classification results. Subsequently, the class assignment of the test subjects was calculated during the test phase. Permutation test can be used to evaluate the probability of obtaining specificity and sensitivity values higher than the ones obtained during the cross-validation procedure by chance. We permuted the labels 100 times, each time randomly assigning the two labels to each image. The whole nested cross-validation process was repeated 5 times, and the final result was the average accuracy of 5 repetitions of the 5-fold cross-validation procedure. Classifier performance was evaluated using basic receiver operating characteristic (ROC) curve. The area under curve (AUC), sensitivity and specificity of the classifier were quantified.

## Statistical Analyses

Comparisons of demographic factors between the two BECTS substates were performed using two-sample *t*-tests. Chi-square (χ^2^) test was used for categorical data. Statistical analysis was performed using IBM SPSS 21.0 version. Data are presented as mean ± standard deviation. All the quoted results are two-tailed values, and *p* < 0.05 was considered as statistically significant.

## Results

### Sample Characteristics

There were no significant differences between the two BECTS substates in mean age (*t* = −1.743, *p* = 0.089), sex (χ^2^ = 0.095, *p* = 0.758) and epilepsy duration (*t* = −1.388, *p* = 0.174). The number of IEDs was (29.71 ± 25.31; range, 4~92) times in the IEDs substate (Table 1).

**Table 1:** Characteristics of BECTS.

	**IEDs**	**Non-IEDs**	***t*-value**	***p*-value**
Mean age, year	8.14 ± 1.88	9.19 ± 2.02	−1.743	0.089
Sex (male, female)	21 (10, 11)	21 (11, 10)	0.095^#^	0.758
Epilepsy duration, month	16.12 ± 16.16	24.66 ± 23.1	−1.388	0.174
Number of IEDs, time	29.71 ± 25.31	N/A	N/A	N/A

Data are mean ± standard deviation values;

# *chi-square value; N/A, Not applicable. BECTS, benign childhood epilepsy with central-temporal spikes; IEDs, interictal epileptiform discharges; N/A, not applicable*.

### Voxel-Based Analysis for Each GCD Metric

**Figure 2:**
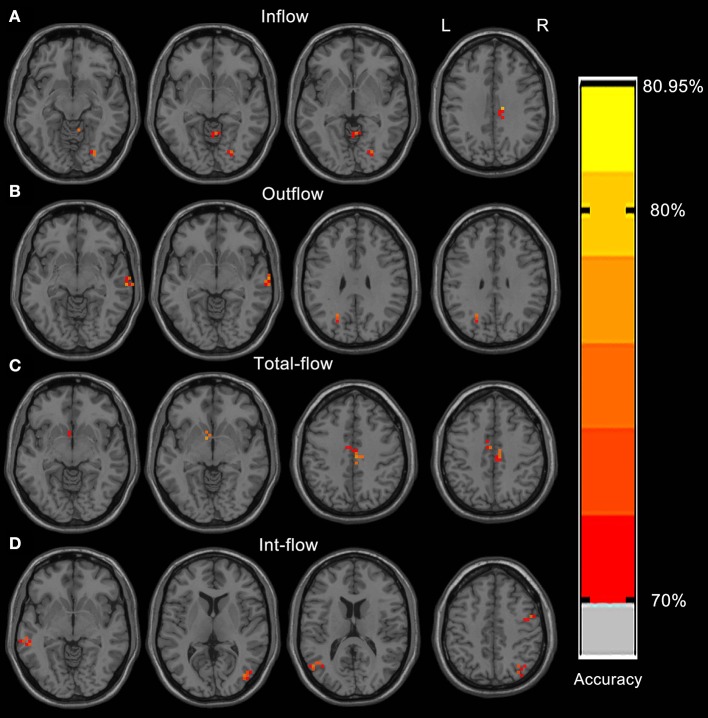
Resulting spatial maps of accuracy for discriminating between IEDs and non-IEDs substates for each of the four GCD metrics. These clusters were identified by setting the threshold of accuracy higher than 70%. Resulting spatial brain areas of accuracy for discriminating between IEDs and non-IEDs substates for Inflow **(A)**, outflow **(B)**, total-flow **(C)**, and int-flow **(D)** connectivity.

The Rolandic area, caudate, dorsal attention network, visual cortex, language networks, and cerebellum showed discriminative effect on distinguishing the IEDs substate from the non-IEDs substate (Figures 2A–D, Table 2). Specifically, the discriminative effect of the Rolandic area was only found in the GCD metric of outflow connectivity (Figure 2B).

**Table 2:** Most important brain regions discriminating between IEDs and non-IEDs substates.

**Conditions**	**Brain regions of peak coordinates**	**R/L**	**BA**	**Peak accuracy (%)**	**MNI coordinates**		
					**X**	**Y**	**Z**
Inflow	Middle occipital gyrus	R	18	78.57	32	−84	−16
Inflow	Cerebellum anterior lobe	R	N/A	73.81	8	−52	−12
Inflow	Cingulate gyrus	R	23, 24	78.57	12	−28	32
Outflow	Middle temporal gyrus	R	21	78.57	64	−20	−8
Outflow	Precuneus	L	7, 19	76.19	−24	−60	32
Total-flow	Caudate head	L	N/A	76.19	−8	4	−4
Total-flow	Cingulate gyrus	L	23, 24	80.95	−8	−12	36
Int-flow	Cerebellum posterior lobe	L	N/A	78.57	−20	−40	−52
Int-flow	Superior temporal gyrus	R	38	80.95	24	12	−40
Int-flow	Middle temporal gyrus	L	21	80.95	−64	−32	−8
Int-flow	Middle occipital gyrus	R	19	76.19	40	−76	8
Int-flow	Middle temporal gyrus	L	39	78.57	−52	−68	20
Int-flow	Precuneus	L	7, 19	73.81	−28	−64	32
Int-flow	Precentral gyrus	R	6	76.19	44	0	40
Int-flow	Superior parietal lobule	R	7	73.81	32	−72	48

*These clusters were identified by setting the threshold of accuracy higher than 70%. IEDs, interictal epileptiform discharges; N/A, not applicable R, right; L, left; BA, Brodmann's area; MNI, Montreal Neurological Institute*.

### Classification Performance

**Figure 3:**
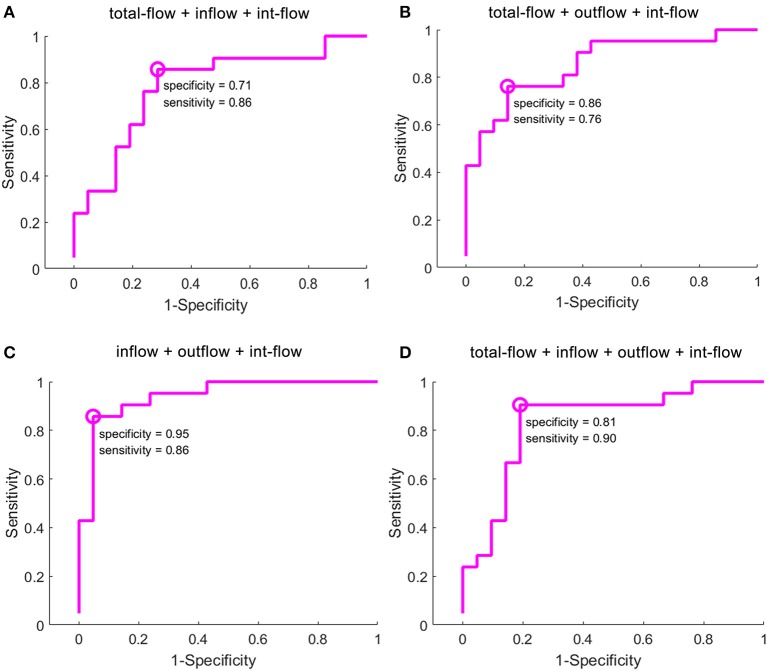
Classification results of GCD maps using selection of the optimal feature dimensions of the SVM-RFE method. GCD, granger causality density; SVM, support vector machine; RFE, recursive feature elimination. The classification accuracy of the combination with total-flow, inflow and int-flow connectivity **(A)**, the combination with total-flow, outflow and int-flow connectivity **(B)**, the combination with inflow, outflow and int-flow connectivity **(C)**, and the combination with total-flow, inflow, outflow and int-flow connectivity **(D)**.

Across the reduced data sets of the evaluated GCD metrics, the combinations with more than three GCD metrics received good classification performances (Figures 3A–D, Table 3). The combination with total-flow, inflow and int-flow connectivity, and the combination with total-flow, outflow and int-flow connectivity did not receive good classification performances (Figures 3A,B). However, the combination with inflow, outflow and int-flow connectivity significantly reached up the classification accuracy and received the best classification performance with the highest accuracy rate (90.83%) and specificity (95%), as well as extremely high AUC value (0.928) and sensitivity (86%) (Figure 3C). Subsequently, the GCD metric of total-flow connectivity entered into the classification and the sensitivity could reach up to 90% (Figure 3D), but the AUC value, accuracy rate and specificity decreased (Table 3). Furthermore, when the functional connectivity density as the input of SVM, poor classification performance was found (sensitivity, 86%; specificity, 48%; Supplementary Figure 1).

**Table 3:** Classification performances using combinations of GCD metrics.

**Classification indicator**	**AUC**	**Accuracy**	**Sensitivity**	**Specificity**
	**value**	**(%)**	**(%)**	**(%)**
Total-flow + inflow	0.703	66.39	62	76
Total-flow + outflow	0.634	66.47	74.67	56
Total-flow + int-flow	0.74	73.61	67	81
Inflow + outflow	0.815	78.61	76	81
Inflow + int-flow	0.9325	75.83	67	95
Outflow+ int-flow	0.8975	83.61	76	90
Total-flow + inflow + outflow	0.675	71.39	62	81
Total-flow + inflow + int-flow	0.758	78.61	86	71
Total-flow + outflow+ int-flow	0.8575	81.11	76	86
Inflow + outflow + int-flow	0.928	90.83	86	95
Total-flow + inflow + outflow + int-flow	0.8175	86.11	90	81

*GCD, granger causality density; IEDs, interictal epileptiform discharges; AUC, area under curve*.

### Classification Capacity

**Figure 4:**
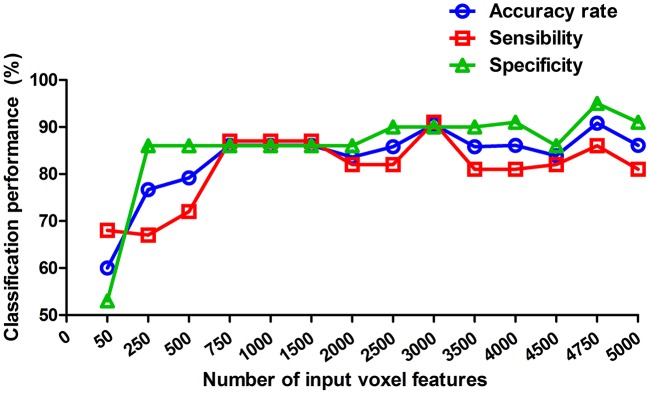
Classification performance at each of reduced data sets. These values reported are of the weighted average of the 5 cross-validation. The reduced data sets were selected by the relief feature selection algorithm. Here, we reported fourteen reduced data sets-50, 250, 500, 750, 1,000, 1,500, 2,000, 2,500, 3,000, 3,500, 4,000, 4,500, 4,750, and 5,000 voxels.

Since the combination with inflow, outflow and int-flow connectivity received the best classification performance, we therefore calculated the classification performance of this combination at each reduced data set to evaluate its stability. Here, we reported fourteen reduced data sets-50, 250, 500, 750, 1,000, 1,500, 2,000, 2,500, 3,000, 3,500, 4,000, 4,500, 4,750, and 5,000 voxels. This combination received good and stable classification performance when the dimension reduced data sets were more than 750 voxels (Figure 4).

## Discussion

BECTS is often associated with clinical syndrome-related specific functional brain impairment (19–24, 28), these impairments not only occur during IEDs substate (29–33), but also non-IEDs substate (33). However, whether the IEDs and non-IEDs substates share the same mechanisms of brain functional impairment remains exploration. The IEDs cannot always be identified by clinical EEG recordings. Thus, the BECTS substate classification has important clinical significance, which may have the potential to early predict IEDs, and assist clinicians for clinical administration. Ji et al. found that both BECTS substates showed consistently abnormal global topology in their functional networks (i.e., decreased global efficiency) relative to that of control subjects, but no differences between the two substates (34). Our study is the first to apply the GCD-SVM model to find a promising model for BECTS substate classification. Our data indicated that the GCD-SVM model achieved extremely high classification performances. Accordingly, although functional connectivity density has been used to characterize abnormal functional connectivity changes in both BECTS substates (35), in the present study, poor performance was observed when the functional connectivity density as the input of the SVM classifier. These findings may suggest that the GCD-SVM model may be served as a sensitive neuroimaging biomarker for BECTS substate classification. In general, the more useful voxel information was entered into the classification procedure, the better the classification performance was. This may explain that: (a) the combinations with more than three GCD metrics could reach up the classification performances relative to single GCD metric; and (b) the classification performance was increased as more numbers of input voxel features (≥750 voxels) were entered into the classification procedure. Specially, some features are uninformative, irrelevant or redundant for classification (36, 37), which may decrease the classification performance. This may explain why the classification performance of the combination with four GCD metrics was decreased relative to the best classification combination. Taken together, our data support our hypothesis.

Machine learning and pattern recognition techniques are being increasingly used in functional MRI data analysis. These methods allow detecting subtle, non-strictly localized effects that may remain invisible to the conventional analysis with univariate statistics (38, 39). In contrast to the conventional analysis, the machine learning technique takes the full spatial pattern of brain activity into account, measures many locations simultaneously, and exploits the inherent multivariate nature of functional MRI data. The use of machine learning algorithm has been applied to discriminate between the newborns with seizures secondary to hypoxic ischemic encephalopathy and those newborns without seizures (40). Furthermore, non-invasive EEG has been used to identify the presence of seizures in pediatric subjects (41). It has been reported that diffusion tensor imaging based SVM classification method has diagnostic advantage over other T1 based classification in temporal lobe epilepsy (42), and appears promising for distinguishing the children with active epilepsy from those with remitted epilepsy or controls with high sensitivity and specificity (43). Our data indicate that the GCD analysis also can be served as a biomarker for BECTS substate classification. In the present study, the combinations with input and output information flow features (inflow + outflow + int-flow connectivity) received the best classification accuracy of 90.83%, which is close to the EEG classification accuracy (44).

There is a close relationship between the hemodynamic changes and brain neural activity. Since the hemoglobin is an oxygen carrier, the neuronal firings may increase the concentration level of local blood oxygen and oxyhemoglobin (antimagnetic), and decrease the deoxygenated hemoglobin (paramagnetic), and therefore change the blood oxygen level dependence (BOLD) signal of regional brain area. This may change the brain excitatory/inhibition balance. It has been reported that the intrinsic spontaneous BOLD signal and the task-induced functional BOLD signal are linearly superimposed (45). This may help us understand why the proposed GCD method (intrinsic spontaneous BOLD signal) has the potential to reflect the IEDs-induced functional BOLD signal. Relative to the non-IEDs substate, the IEDs-related activation may increase the input and/or output information flow connectivity of the epilepsy-related brain networks, and change the brain network connectivity architecture (edge and/or directions). Consistently, Zhu et al. found that the mapped features of the resting-state functional MRI could distinguish the two BECTS substates (46). These findings may support the high classification performance of the GCD-SVM model in distinguishing the two BECTS substates. The brain regions of the Rolandic area, caudate, dorsal attention network, visual cortex, language networks, and cerebellum, exhibiting high discriminating value, may be the neurobiological base of the high classification accuracy between the two BECTS substates. These findings suggest that the proposed GCD-SVM model may be helpful for BECTS substate classification by exploiting multitype and multidimensional voxel features with discriminating value.

## Conclusions

To summarize, the proposed GCD-SVM model could be served as a potential neuroimaging biomarker to discriminate between the two BECTS substates, which may expand our understanding of the neurobiological mechanism of BECTS. The performance of the GCD-SVM model has the potential to assist clinicians for early diagnosis, clinical administration, and monitoring the efficacy of disease-modifying therapies. This may promote the clinical management of BECTS.

The strengths of our study are the performance of innovative GCD–SVM method and the invaluable data of the IEDs substate. However, there are several potential limitations that should be noted. First, varying quality of preictal data for different subjects may obtain varying prediction performances. Therefore, small sample size and single center data limited its generality. A larger number of sample sizes and multiple center studies are necessary to corroborate our findings. Second, the small number of IEDs may limit the classification performance. The IEDs substate with more numbers of IEDs may increase the classification accuracy. Third, different types and the density (i.e., the IED number for window length) of the IEDs were not addressed in the present study. Fourth, traditionally, BOLD signal in the white matter (WM) was regarded as noise and was regressed out in the preprocessing step in our study. However, recent research showed that the WM signal was also biologically meaningful. It has structural basis and could be modulated by cognitive state (47). In neurological disease, such as PD, it is of great significance in clinical application (48). In this study, we regressed out the WM signal in the preprocessing step because of unclear biological mechanism. Fifth, the data of non-IEDs and IEDs substates were came from different subjects. However, 8 min MRI scan time are not enough to obtain enough data to divide the data of one subject into non-IEDs and IEDs substates. Therefore, it is equally important to classify the non-IEDs and IEDs substates from different subjects for MRI data. Sixth, twenty BECTS (10 IEDs, 10 non-IEDs) were not first-time visitors and had taken medication before. In the present study, the medication effects were not taken into account.

## Data Availability Statement

All datasets analyzed for this study can be found in the article/Supplementary Material.

## Author Contributions

XJ-D, ZZ, and GL conceived and designed the whole experiment. XJ-D, QZ, and YX collected the data. XJ-D and QX take responsibility for the integrity of the data, the accuracy of the data analysis, and the statistical data analysis. XJ-D wrote the main manuscript text and under took the critical interpretation of the data. All authors contributed to the final version of the paper and have read, as well as approved the final manuscript.

### Conflict of Interest

The authors declare that the research was conducted in the absence of any commercial or financial relationships that could be construed as a potential conflict of interest.

## Supplementary Material

Supplementary Figure 1 Classification results of functional connectivity density.
