# Supplementary figures and images for: BECTS Substate Classification by Granger Causality Density Based Support Vector Machine Model
Source: Front Neurol. 2019 Nov 14;10:1201. doi: 10.3389/fneur.2019.01201 (PMC6868120; doi:10.3389/fneur.2019.01201)

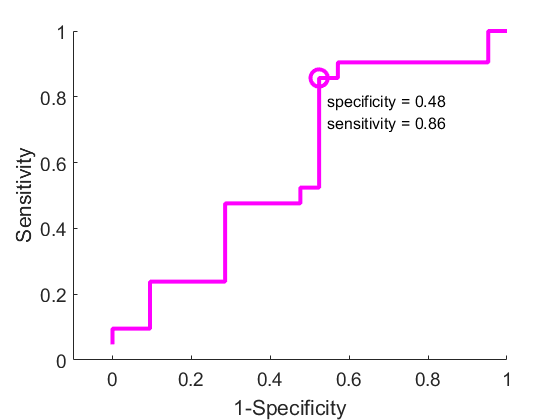

Supplement: Supplementary Figure 1 — Classification results of functional connectivity density. [file Image_1.TIF]
